# Supplementary material for: Simultaneous Metabarcoding and Quantification of Neocallimastigomycetes from Environmental Samples: Insights into Community Composition and Novel Lineages
Source: Microorganisms. 2022 Aug 30;10(9):1749. doi: 10.3390/microorganisms10091749 (PMC9504928; doi:10.3390/microorganisms10091749)
Supplement: Supplementary file 1 [file microorganisms-10-01749-s001.zip › 1_Suplementary Data S1.pdf]

Supplementary Data S1: Primers targeting the D2 region from the 28S large subunit designed using Primrose 2.17 and tested in silico to determine their specificity for Neocallimastigomycetes.

Primer	Sequence 5´-> 3´
1_69489_57C	CCTTKACGAATTGTAGTTTAWAGA
2_71389_59C	GATACACTTTCAAMGAGUCGGA
3_72987_58C	GCGTTTGACACCAGTGTTGT
4_739344_57C	ATGCACTTTTCRKTTAACAAGTC
5_76533_52C	YATAGACAACTTGTTGACTAT
6_74895_57C	TGAGGHCWGCAGCGTARA
7_GGNL1F_57C	CATAGAGGGTGAGAATCCCGTA
8_GGNL1R_56C_modif	TACCTACGCTTAGGATGTTGA
9_AF-LSU rev 1.3.1._45-60C modif	GCACTTYTYRKKKWACAAGTC
