# Supplementary material for: Simultaneous Metabarcoding and Quantification of Neocallimastigomycetes from Environmental Samples: Insights into Community Composition and Novel Lineages
Source: Microorganisms. 2022 Aug 30;10(9):1749. doi: 10.3390/microorganisms10091749 (PMC9504928; doi:10.3390/microorganisms10091749)
Supplement: Supplementary file 1 [file microorganisms-10-01749-s001.zip › 2_Supplementary Data S2 text.pdf]

Supplementary Data S2. Abundance of clones (Sanger sequenced) and ASV reads (Illumina sequenced) displaying the *Neocallimastigomycetes* community in the 10 fecal samples.
