# Supplementary material for: Simultaneous Metabarcoding and Quantification of Neocallimastigomycetes from Environmental Samples: Insights into Community Composition and Novel Lineages
Source: Microorganisms. 2022 Aug 30;10(9):1749. doi: 10.3390/microorganisms10091749 (PMC9504928; doi:10.3390/microorganisms10091749)
Supplement: Supplementary file 1 [file microorganisms-10-01749-s001.zip › 3_Suplementary Data S2 Table ASV-Clones.pdf]

Taxonomy according to D1-D2/LSU phylogenetic analysis	ASV	Cow		Lama		Elephant		Mara		Horse	Alpine Ibex	Alpaca	Kangaroo	Bison	Giraffe	Total read counts	Total clone count
		reads	Sanger clones	reads	Sanger clones	reads	Sanger clones	reads	Sanger clones	reads	reads	reads	reads	reads	reads		
Neocallimastix frontalis	ASV0001	829		484	1	32					1897	2594		351		6187	1
Neocallimastix frontalis	ASV0002	564														564	0
Neocallimastix frontalis	ASV0003	2578	6			941	3				4181					7700	9
Neocallimastix frontalis	ASV0004	444	1													444	1
Neocallimastix frontalis	ASV0005	2461	1	23		232	2				273	486		81		3556	3
Neocallimastix frontalis	ASV0006	97														97	0
Neocallimastix frontalis	ASV0007	336				361	2									697	2
Neocallimastix frontalis	ASV0008	1225	1													1225	1
Neocallimastix frontalis	ASV0009	2302	2	444		935	4			62	1713	11383		1		16840	6
Neocallimastix frontalis	ASV0010													293		293	0
Neocallimastix frontalis	ASV0011	250	1 (98.1%)	6		486	1				130	260		762		1894	2
Neocallimastigaceae unclassified	ASV0012											177				177	0
Orpinomyces intercalaris	ASV0013			1906	2							291	24			2221	2
Orpinomyces intercalaris	ASV0014			58	1											58	1
Orpinomyces joyonii	ASV0015			59								46				105	0
Orpinomyces intercalaris	ASV0016			427	2											427	2
Orpinomyces intercalaris	ASV0017			716	3											716	3
Orpinomyces intercalaris	ASV0018													106		106	0
Orpinomyces joyonii	ASV0019													482		482	0
Orpinomyces joyonii	ASV0020			448								783		5		1236	0
Orpinomyces joyonii	ASV0021													178		178	0
Orpinomyces joyonii	ASV0022													73		73	0
Orpinomyces joyonii	ASV0023			165	1							745		492		1402	1
Orpinomyces joyonii	ASV0024													1872		1872	0
Orpinomyces joyonii	ASV0025			93	1									124		217	1
Orpinomyces joyonii	ASV0026											194				194	0
Orpinomyces joyonii	ASV0027			932	4							1461				2393	4
Orpinomyces joyonii	ASV0028									25						25	0
Piromyces communis	ASV0030										716					716	0
Piromyces sp. 4	ASV0031	1191	1													1191	1
Piromyces sp. 6	ASV0032					15										15	0
Piromyces sp. 6	ASV0033		1		1	3466	7<br>3 (98.73%)									3466	12
Cand. Piromyces potentiae	ASV0034											440				440	0
Cand. Piromyces potentiae	ASV0035			24												24	0
Cand. Piromyces potentiae	ASV0036			282	1							313	4			599	1
Cand. Piromyces potentiae	ASV0037													75		75	0
Cand. Piromyces potentiae	ASV0038	60														60	0
Cand. Piromyces potentiae	ASV0039	481														481	0
Cand. Piromyces potentiae	ASV0040		5			3155	13<br>1 (98.73%)									3155	19
Neocallimastigaceae genus 23 sp. 1	ASV0041	699		214	1						500	657		116		2186	1
Neocallimastigaceae genus 23 sp. 1	ASV0042	96	1													96	1
Neocallimastigaceae genus 23 sp. 1	ASV0043			257								926		935		2118	0
Neocallimastigaceae genus 23 sp. 1	ASV0044	487	4								70					557	5
Neocallimastigaceae genus 23 sp. 1	ASV0045			231										390		621	0
Neocallimastigaceae genus 23 sp. 1	ASV0046													1		1	0

Taxonomy according to D1-D2/LSU phylogenetic analysis	ASV	Cow		Lama		Elephant		Mara		Horse	Alpine Ibex	Alpaca	Kangaroo	Bison	Giraffe	Total read counts	Total clone count
		reads	Sanger clones	reads	Sanger clones	reads	Sanger clones	reads	Sanger clones	reads	reads	reads	reads	reads	reads		
Neocallimastigaceae genus 23 sp. 2	ASV0047											366				366	0
Neocallimastigaceae genus 23 sp. 3	ASV0048											453		296		749	0
Neocallimastigaceae genus 23 sp. 3	ASV0049			396	1							391				787	1
Cyllamyces aberensis	ASV0050													1502		1502	0
Cyllamyces unclassified	ASV0051													29		29	0
Cyllamyces unclassified	ASV0052													344		344	0
Cyllamyces unclassified	ASV0053													68		68	0
Cyllamyces unclassified	ASV0054													83		83	0
Cyllamyces unclassified	ASV0055													422		422	0
Cyllamyces unclassified	ASV0056			83												83	0
Cyllamyces unclassified	ASV0057			113	3											113	3
Cyllamyces unclassified	ASV0058											132				132	0
Cyllamyces unclassified	ASV0059													199		199	0
Cyllamyces unclassified	ASV0060													1114		1114	0
Cyllamyces unclassified	ASV0061			32								298		5647		5977	0
Cyllamyces unclassified	ASV0062			137												137	0
Cyllamyces unclassified	ASV0063													1181		1181	0
Cyllamyces unclassified	ASV0064													2955		2955	0
Cyllamyces unclassified	ASV0065													197		197	0
Cyllamyces unclassified	ASV0066													40		40	0
Cyllamyces unclassified	ASV0067													200		200	0
Cyllamyces unclassified	ASV0068													7		7	0
Cyllamyces unclassified	ASV0069											116				116	0
Cyllamyces unclassified	ASV0070			254										2101		2355	0
Cyllamyces unclassified	ASV0071													546		546	0
Cyllamyces unclassified	ASV0072													147		147	0
Cyllamyces unclassified	ASV0073													44		44	0
Cyllamyces unclassified	ASV0074													483		483	0
Cyllamyces unclassified	ASV0075													36		36	0
Cyllamyces unclassified	ASV0076													80		80	0
Cyllamyces unclassified	ASV0077													19		19	0
Cyllamyces unclassified	ASV0078													192		192	0
Caecomyces churrovis	ASV0079													787		787	0
Caecomyces churrovis	ASV0080			299								851				1150	0
Caecomyces churrovis	ASV0081	621		193						21	8863	621				10319	0
Caecomyces churrovis	ASV0082	15791	13												1693	17484	14
Caecomyces churrovis	ASV0083					6296	15<br>2 (98.73%)									6296	17
Caecomyces communis	ASV0084						1				10880					10880	1
Paucimyces sp. 2	ASV0085			46												46	0
Paucimyces sp. 2	ASV0086			605										169		774	0
Feramyces austinii	ASV0087														99	99	0
Feramyces austinii	ASV0088														9589	9589	0
Feramyces austinii	ASV0089														816	816	0
Feramyces austinii	ASV0090														3785	3785	0
Feramyces austinii	ASV0091														104	104	0
Feramyces austinii	ASV0092														13	13	0
Feramyces austinii	ASV0093														1058	1058	0

Taxonomy according to D1-D2/LSU phylogenetic analysis	ASV	Cow		Lama		Elephant		Mara		Horse	Alpine Ibex	Alpaca	Kangaroo	Bison	Giraffe	Total read counts	Total clone count
		reads	Sanger clones	reads	Sanger clones	reads	Sanger clones	reads	Sanger clones	reads	reads	reads	reads	reads	reads		
Feramyces austinii	ASV0094														3281	3281	0
Feramyces austinii	ASV0095														13408	13408	0
Neocallimastigaceae genus 24 unclassified	ASV0096											824				824	0
Neocallimastigaceae genus 24 unclassified	ASV0097										856					856	0
Neocallimastigaceae genus 24 unclassified	ASV0098										436					436	0
Neocallimastigaceae genus 24 unclassified	ASV0099			103												103	0
Neocallimastigaceae genus 24 unclassified	ASV0100			477												477	0
Neocallimastigaceae genus 24 unclassified	ASV0101			54												54	0
Neocallimastigaceae genus 24 unclassified	ASV0102			277												277	0
Neocallimastigaceae genus 25 unclassified	ASV0103											228		3871		4099	0
Neocallimastigaceae genus 25 unclassified	ASV0104											181		2442		2623	0
Anaeromyces mucronatus	ASV0105											263				263	0
Anaeromyces mucronatus	ASV0106			853	1							400				1253	1
Anaeromyces mucronatus	ASV0107											405				405	0
Anaeromyces mucronatus	ASV0108			307												307	0
Anaeromyces mucronatus	ASV0109											83				83	0
Anaeromyces mucronatus	ASV0110			909	1											909	1
Anaeromyces mucronatus	ASV0111			624	2							68				692	2
Anaeromyces mucronatus	ASV0112													187		187	0
Anaeromyces mucronatus	ASV0113													148		148	0
Anaeromyces mucronatus	ASV0114											60	24			84	0
Neocallimastigaceae genus 21 unclassified	ASV0115			521								76				597	0
Neocallimastigaceae genus 21 unclassified	ASV0116										185					185	0
Neocallimastigaceae genus 26 unclassified	ASV0117										19		26079			26098	0
Neocallimastigaceae genus 26 unclassified	ASV0118												146			146	0
Neocallimastigaceae genus 26 unclassified	ASV0119													58		58	0
Neocallimastigaceae genus 22 unclassified	ASV0120							13852	34<br>1<br>(98.73%)							13852	35
Neocallimastigaceae genus 22 unclassified	ASV0121							6095	7							6095	7
Neocallimastigaceae genus 22 unclassified	ASV0122							5619	11							5619	11
Khoyollomyces ramosus	ASV0123									2903						2903	0

Taxonomy according to D1-D2/LSU phylogenetic analysis	ASV	Cow		Lama		Elephant		Mara		Horse	Alpine Ibex	Alpaca	Kangaroo	Bison	Giraffe	Total read counts	Total clone count
		reads	Sanger clones	reads	Sanger clones	reads	Sanger clones	reads	Sanger clones	reads	reads	reads	reads	reads	reads		
Khoyollomyces ramosus	ASV0124					99				11645						11744	0
Khoyollomyces ramosus	ASV0125									203						203	0
Khoyollomyces ramosus	ASV0126									1891						1891	0
Khoyollomyces ramosus	ASV0127									103						103	0
Khoyollomyces ramosus	ASV0128									521						521	0
Khoyollomyces ramosus	ASV0129									4172						4172	0
Khoyollomyces ramosus	ASV0130									2604						2604	0
Total count	129	30512	37	13052	26	16018	54	25566	53	24150	30719	26572	26277	31931	33846	258643	
ASVs per sample		18	12	37	16	11	9	3	3	11	15	34	5	50	10		
Clone total count																	172
