# Supplementary material for: Simultaneous Metabarcoding and Quantification of Neocallimastigomycetes from Environmental Samples: Insights into Community Composition and Novel Lineages
Source: Microorganisms. 2022 Aug 30;10(9):1749. doi: 10.3390/microorganisms10091749 (PMC9504928; doi:10.3390/microorganisms10091749)
Supplement: Supplementary file 1 [file microorganisms-10-01749-s001.zip › 4_Supplementary Data S3 text.pdf]

Supplementary Data S3. Fully resolved phylogenetic tree of the AGF LSU D1-D2 region. DQ536493 (*Chytriomyces* sp. WB235A) was included as an outgroup. Filled symbols: reference strain(s) of a validly published species is available; valid taxonomic assignments are in italics. Open symbols: No cultured reference strain available, species defined only by genetic traits („genospecies"). Filled triangles pointing left: compressed clade. Triangle pointing down: Order level node. Triangle pointing up: Potential family level node. Square: Genus level node (established or > 95% sequence identity); „>" in assignments means that the clades have genus level but only one species can be shown. Circle: Species-level node (established or > 98.5 % sequence identity).
