# Supplementary material for: Simultaneous Metabarcoding and Quantification of Neocallimastigomycetes from Environmental Samples: Insights into Community Composition and Novel Lineages
Source: Microorganisms. 2022 Aug 30;10(9):1749. doi: 10.3390/microorganisms10091749 (PMC9504928; doi:10.3390/microorganisms10091749)
Supplement: Supplementary file 1 [file microorganisms-10-01749-s001.zip › 6_Supplementary Data S4 text.pdf]

Supplementary Data S4. Fully resolved phylogenetic tree with the AGF-LSU-EnvS (D2 LSU) amplicons of the current study. DQ536493 (*Chytriomyces* sp. WB235A) was used as an outgroup. Accession numbers ON819131 - ON819177 are from Joshi *et al.* [25].
