# Supplementary figures and images for: Simultaneous Metabarcoding and Quantification of Neocallimastigomycetes from Environmental Samples: Insights into Community Composition and Novel Lineages
Source: Microorganisms. 2022 Aug 30;10(9):1749. doi: 10.3390/microorganisms10091749 (PMC9504928; doi:10.3390/microorganisms10091749)

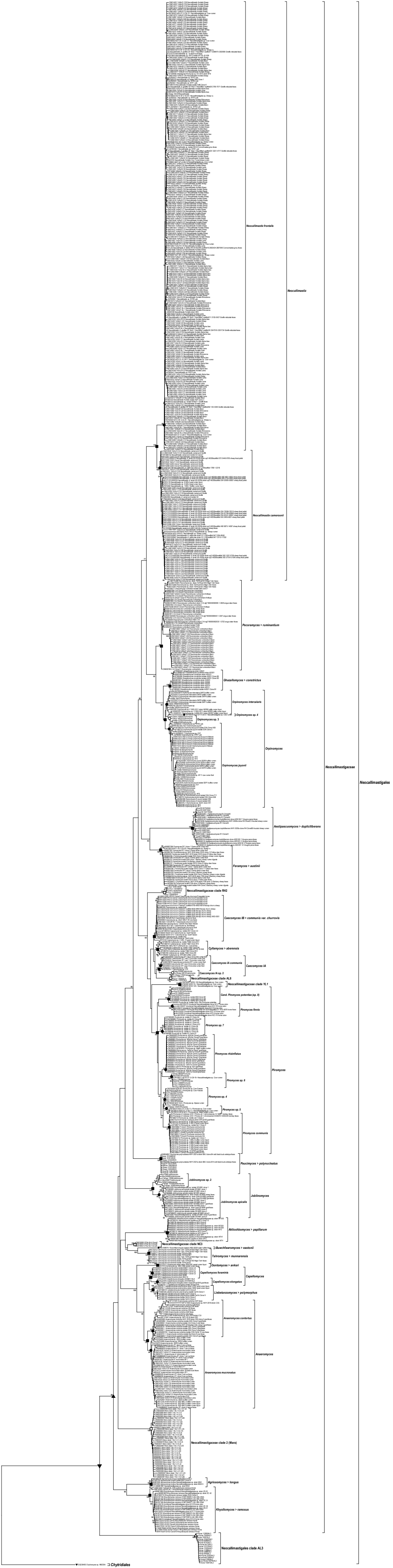

Supplement: Supplementary file 1 [file microorganisms-10-01749-s001.zip › 5_Supplementary Data S3 D1-D2 complete.pdf]

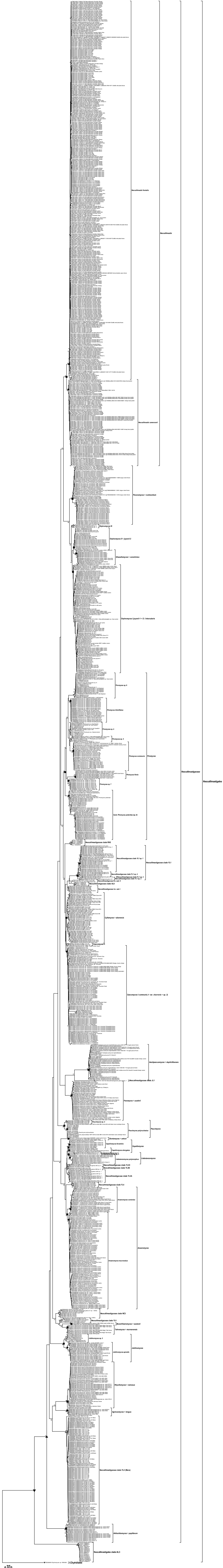

Supplement: Supplementary file 1 [file microorganisms-10-01749-s001.zip › 7_Supplementary Data S4 D2 complete.pdf]
